# Supplementary material for: Unsupervised determination of lung tumor margin with widefield polarimetric second-harmonic generation microscopy
Source: Sci Rep. 2022 Dec 1;12:20713. doi: 10.1038/s41598-022-24973-1 (PMC9715953; doi:10.1038/s41598-022-24973-1)
Supplement: Supplementary file 1 — Supplementary Information. [file 41598_2022_24973_MOESM1_ESM.pdf]

Supplementary Document: Unsupervised determination of lung tumor margin with widefield polarimetric second-harmonic generation microscopy

Supplementary Note: Validation of K-Means of PC2 on additional areas

K-Means clustering is a powerful technique, as it enabled segmentation of the data into groups with common trends and behaviors [1, 2, 3]. In this study, K-Means clustering was applied to widefield P-SHG images acquired from a large extended region of non-small cell lung carcinoma sample, encompassing both tumor and normal tissues. It was found that principal components analysis (PCA) improved the K-Means performance, resulting in higher silhouette scores, as shown in Fig. 3 of the manuscript. More importantly, it was evident that K-Means applied to the second principal component (PC2) identified the location of the tumor margin, suggesting potential applications of the methodology in clinical studies and diagnostics (see Fig. 4 of the manuscript). However, it is vital to investigate additional regions and samples to assess the reproducibility of the findings.

A total of 9 additional lung tissue regions across 2 additional sample slides, each belonging to different patients (3 patient samples overall, including the large extended region), were measured to validate the applicability of PC2 in tumor margin detection. Following the P-SHG analysis of the images, the computed polarimetric and texture parameters were used to form a linear combination corresponding to PC2 found from the large extended region presented in the manuscript. This procedure was performed by extracting the parameter coefficients of the PC2 from the large extended region, and subsequent application of the coefficients on the parameters found for the additionally imaged areas. Having recreated the PC2 for all imaged areas, K-Means was used to identify the binary, silhouette, continuous, and median filtered maps, as described in the manuscript. As such, the results may be used to directly validate the reproducibility of the PC2 findings in identifying the tumor margin.

Supplementary Fig. 1 shows the results of the analysis, similar to the format shown in Fig. 3 of the manuscript. It is clear that seemingly normal tissue regions are highlighted in yellow, while the areas in close proximity of tumor are depicted in magenta, corresponding to the trends observed in the large extended region presented in Fig. 3 of the manuscript.

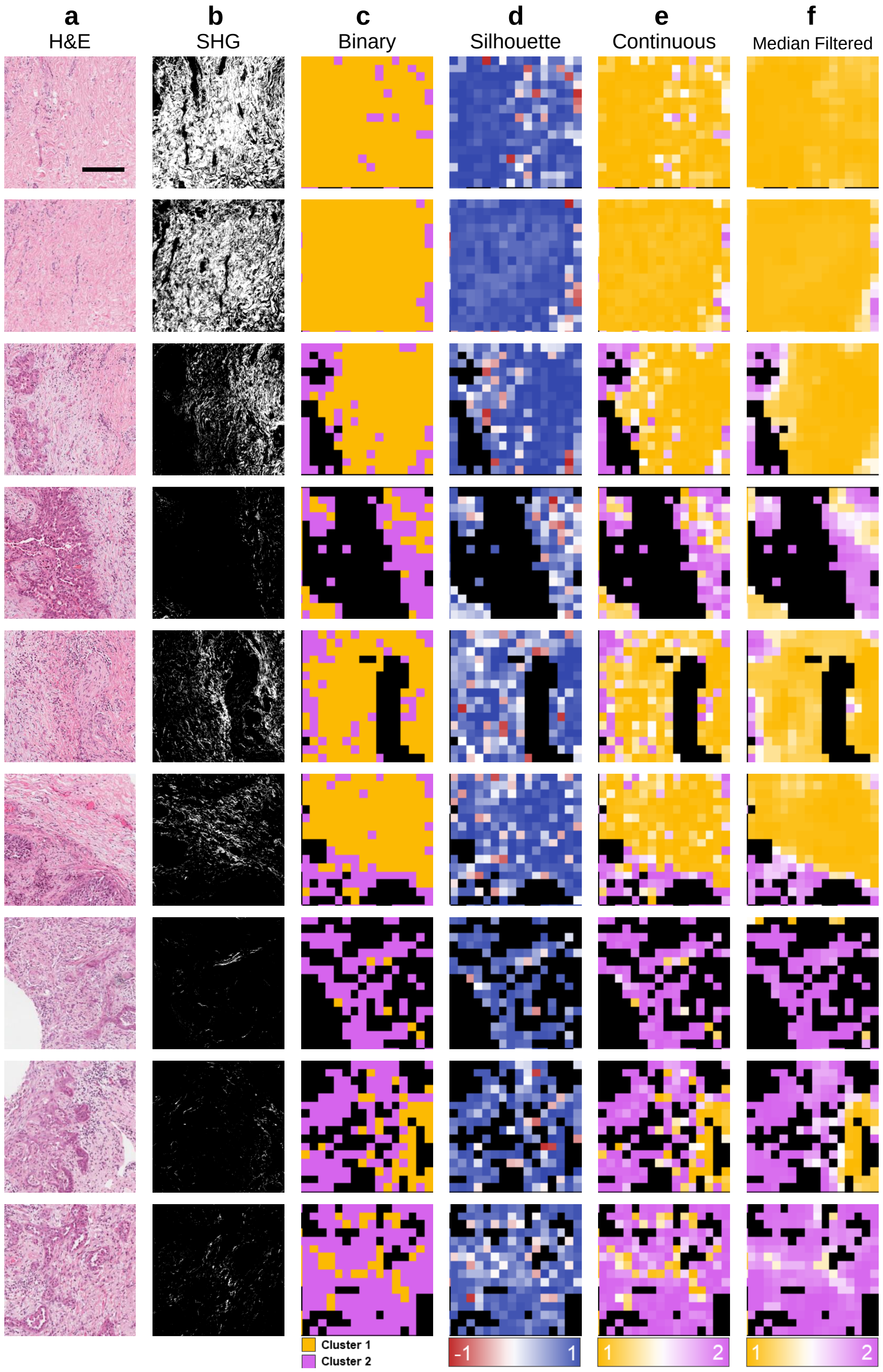

Figure 1: K-Means clustering with PC2. A total of 9 additional areas from 3 sample slides each belonging to different patients, were imaged to further investigate K-Means clustering with PC2. a, H&E brightfield images of the tissue regions. b, SHG intensity images show the expected reduction of signal near tumor. c, Binary maps result from application of K-Means clustering on the polarimetric and texture parameters corresponding to PC2 of the large extended region in the manuscript. d, Silhouette map highlights the performance of clustering for each sub-image. e, Continuous map combines the binary and silhouette score maps to create a single map of the clusters ranging from 1 (perfectly belonging to cluster 1) to 2 (perfectly belonging to cluster 2). f, A median filter is applied on the continuous map to create a smooth image for better visualization. Scale bar: 200μm

Nonetheless, it is important to compare the clustering results with ground-truth labels, as indicated by an expert pathologist. Supplementary Fig. 2 depicts the side-by-side comparison of the continuous map with ground-truth maps, similar to Fig. 4 of the manuscript. It is evident from Supplementary Figs. 2a-i, that most regions belonging to the yellow cluster are also associated with the pathologist annotations of the normal region in blue. Similarly, areas

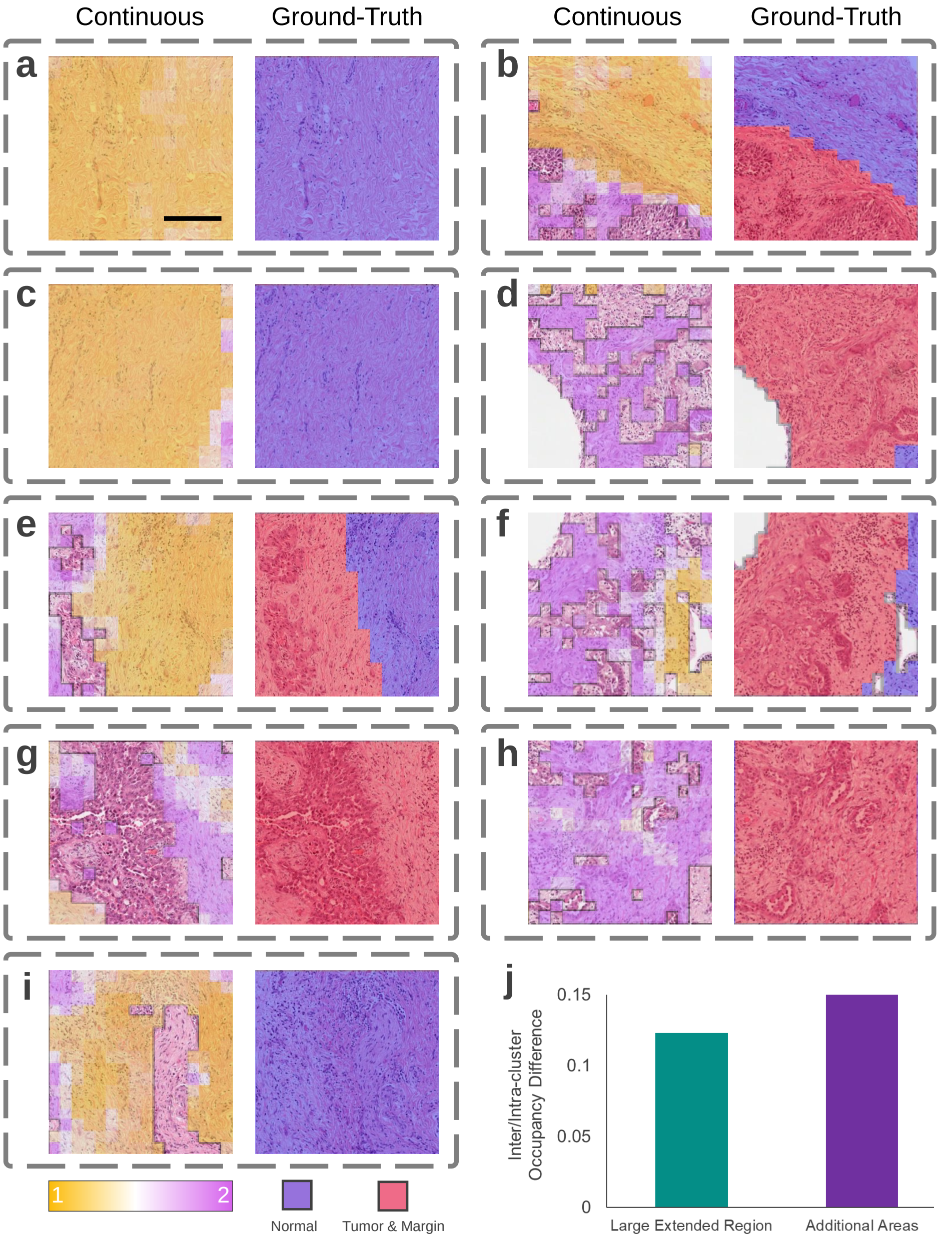

Figure 2: Tumor margin identification with PC2. Continuous cluster and ground-truth maps provided by an expert pathologist are overlaid the H&E images and compared across 9 different tissue regions (a-i). Cluster 1 in yellow corresponds well with the normal tissue in blue, while cluster 2 in magenta matches well with the tumor regions in red. j, The inter/intracluster Occupancy Difference is computed and compared with the large extended region in the manuscript. It is evident that IIOD of the additional areas is larger than the large extended region in the manuscript, thus, suggesting strong reproducibility and usefulness of the results. Scale bar: $200\mu m$

highlighted in magenta cluster mostly correspond with pathologist annotations of tumor tissue in red. Furthermore, the inter/intracluster Occupancy Difference (IIOD) computed for the additional areas (0.16) is in fact larger than the large extended region (0.12), as shown in Supplementary Fig. 2j. Larger values of IIOD signify better separation of the clusters across the normal and tumor annotated regions (see Methods section of the manuscript for details). Thus, the results provide strong support for reproducibility and usefulness of the PC2 and K-Means clustering in identifying tumor margin.

Supplementary Note: Optimal number of sub-images for K-Means

An extended large region of non-small cell lung carcinoma tissue was imaged with the widefield P-SHG microscope [4], and the polarimetric parameters were extracted. In order to generate high-resolution maps of texture parameters, including contrast, correlation, entropy, angular second moment, and inverse difference moment [5], the underlying region of interest was first subdivided into sub-images. Furthermore, the polarimetric and texture parameters of subdivided area were analyzed with PCA and K-Means clustering to highlight morphological changes in the collagenous extracellular matrix, and ultimately identify the tumor margin. Therefore, it is important to assess the effect of subdivision level on the performance of the clustering algorithm.

To identify the optimal subdivision level for the analysis, polarimetric parameters in each of the 12 images used to tile the extended region were subdivided into 1, 4, 16, 64, 256, 1024, 4096, and 16384 sub-images. Following the subdivision, a binary K-Means clustering was applied and the silhouette

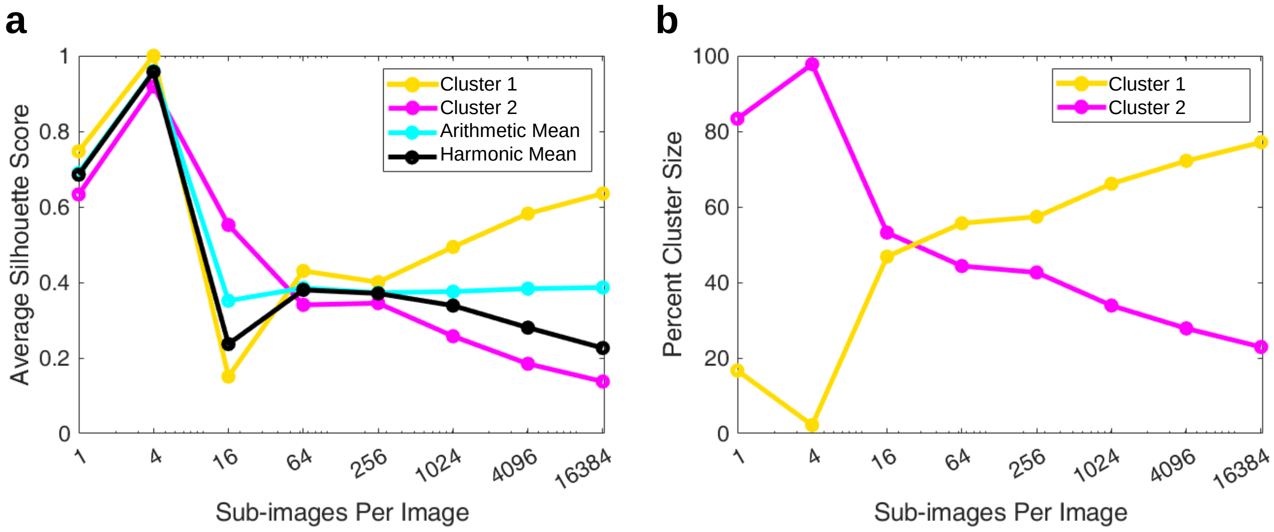

Figure 3: Subdivision level optimization. a, Arithmetic average silhouette scores of clusters 1 (yellow) and 2 (magenta), along with the arithmetic (cyan) and harmonic (black) means of the combination of clusters were computed at various subdivision levels, corresponding to the large extended region of interest. The harmonic mean captures the decrease in silhouette score of cluster 2, resulting in optimal subdivision levels at 64 and 256 sub-images per image. b, Percent cluster size shows that at low and high subdivision levels, K-Means forms predominantly a single cluster, which could result in artificially increased silhouette scores.

scores of each individual cluster, along with the arithmetic and harmonic means of the silhouette scores were computed [6]. Supplementary Fig. 3a illustrates the resulting silhouette scores at all considered subdivisions.

It is evident that clustering with 1 or 4 sub-image per image, resulted in the larger silhouette scores across the board. However, when considering the percentage of clusters found in the data (Supplementary Fig. 3b), it is evident that mostly a single cluster (cluster 2) is formed. Moreover, at such low subdivision levels, the details underpinning more than 50 million pixels in the extended imaged region are disregarded and mapping the features across the area is ineffective. Thus, we remove 1 and 4 sub-images per image from the list of potential subdivision levels.

It is clear that at subdivisions of 16 and larger, the percent cluster sizes are more comparable and the silhouette scores are more stable. As the subdivision level increases, the disparity between cluster sizes increases, and the algorithm once again effectively forms a single cluster. This effect is not reflected in the typically used arithmetic mean of the silhouette scores, as it tends to plateau around silhouette score of 0.4 (Supplementary Fig. 3a). We notice that as the subdivision level increases, the performance of cluster 2 suffers, as indicated by the decreasing magenta curve, while the opposite is true for cluster 1. To fully capture this effect, harmonic mean of silhouette scores from clusters 1 and 2 were computed, similar to the F1-Score used to combine precision and recall in supervised machine learning. As depicted by the black curve in Supplementary Fig. 3a, the harmonic mean indeed penalizes the decrease in performance at larger subdivisions due to cluster size disparity, resulting in two optimal subdivision levels at 64 and 256 sub-images per image. Since the aim of this investigation is to provide a detailed and high-resolution map of morphological changes of the collagenous extracellular matrix across the tissue, it is ideal to use larger subdivision. As such, 256 sub-images per image were used to subdivide the data for further investigations.

References

[1] Zheng, B., Yoon, S., & Lam, S. Breast cancer diagnosis based on feature extraction using a hybrid of K-Means and support vector machine algorithms. *Expert Syst. Appl.* **41**(4), 1476-1482 (2004).

[2] Anas, M., Gupta, K., & Ahmad, S. Skin cancer classification using K-means clustering. *Int. j. eng. res* **5**(1), 62-65 (2017).

[3] Nidheesh, N., Nazeer, K. A., & Ameer, P. M. An enhanced deterministic K-Means clustering algorithm for cancer subtype prediction from gene expression data. *Comput. Biol. Med.* **91**, 213-221 (2017).

[4] Mirsanaye, K. et al. Machine learning-enabled cancer diagnostics with widefield polarimetric second-harmonic generation microscopy. *Sci. Rep.*. **12**, 10290 (2022).

[5] Haralick R.M., Shanmugam K. & Dinstein Its'hak. Textural features for image classification. *IEEE Trans. Syst. Man Cybern.* **SMC-3**, 610-621 (1973).

[6] Rousseeuw, P. J. Silhouettes: a graphical aid to the interpretation and validation of cluster analysis. *J. Comput. Appl. Math.* **20**, 53-65 (1987).
